# Resource allocation to pea plant nodules impacted by nitrogen fixation potential of infecting rhizobia

**DOI:** 10.1093/ismejo/wrag097

**Published:** 2026-04-17

**Authors:** Thomas J Underwood, Philip S Poole

**Affiliations:** Department of Biology, Life and Mind Building, South Parks Rd, University of Oxford, Oxford OX1 3EL, United Kingdom; Department of Biology, Life and Mind Building, South Parks Rd, University of Oxford, Oxford OX1 3EL, United Kingdom

**Keywords:** sanctioning, rhizobia, legume, symbiosis, nodules

## Abstract

Legumes host nitrogen-fixing bacteria, called rhizobia, within specialized root structures called ‘nodules’, where carbon from the plant is exchanged for ammonia fixed from N_2_ by the bacteria. Legumes can host multiple bacterial strains at the same time, which vary in their fixation effectiveness, but legumes sanction nodules containing less effectively fixing strains by reducing the provision of nutrients. Understanding how sanctions are applied by plants and how bacteria may try to avoid them is important for understanding the stability of legume–rhizobial symbioses. Using near isogenic *Rhizobium leguminosarum* strains, on pea, we demonstrate that sanctions are sensitive to the proportion of nodules occupied by a less effective strain and by using split roots we show that sanctions are applied based on a global comparison of nodules across the plant’s root system. By using several rhizobia with different levels of fixation, but all derived from the same parent, we show that pea plants can differentiate between bacteria with relatively small variations in fixation effectiveness. We demonstrate that peas integrate global signals to determine whether individual nodules are sanctioned. At the same time these results show that poorly fixing strains can avoid sanctions if they dominate nodulation.

## Introduction

Legumes, such as *Pisum sativum* (pea), can establish a mutualistic symbiosis with nitrogen-fixing bacteria known as ‘rhizobia’ [1]. In nitrogen-limited environments, this association allows the host plant to access a biologically usable form of nitrogen. Central to this symbiosis is the formation of root nodules, specialized organs within which rhizobia differentiate into bacteroids capable of fixing atmospheric nitrogen into ammonia [2]. In exchange for fixed nitrogen, the host supplies carbon, primarily in the form of C4-dicarboxylates. This reciprocal exchange of nutrients underpins the mutualistic nature of the interaction [3].

Nodules provide a highly advantageous niche for rhizobia, enabling them to multiply to high densities before being released back into the soil upon nodule senescence [4]. However, this ecological opportunity opens the door for less effective ‘cheater’ strains: rhizobia that colonize nodules and benefit from carbon and amplification of numbers while investing less into nitrogen fixation than other strains [5].

The symbiosis between pea and *Rhizobium leguminosarum* bv. viciae Rlv3841 is highly specific. *Pisum sativum* is only nodulated by a narrow set of rhizobia, and primarily *R. leguminosarum*. A complex signalling pathway ensures only these compatible bacteria elicit nodule formation by the host plant [1]. Signalling begins with plants releasing flavonoids that induce rhizobia to produce lipochitooligosaccharides (LCOs), also known as ‘nod factors’. Nod factors signal the presence of the rhizobia to the plant, triggering a signalling cascade resulting in rhizobial attachment and root hair curling, which trap rhizobia [6]. Therefore, infection and nodule formation occur both before rhizobia are intracellularly accommodated by plant cells and before they differentiate into N_2_ fixing bacteroids. The ability of bacteria to induce nodule formation is spatially and temporally separated from their ability to fix N_2_. As such, poorly fixing or nonfixing strains (cheaters) may still be competitive for inducing nodulation by their cognate host legume. This raises the question as to how host plants select against less effective strains.

Previous studies [7–11] have demonstrated that host plants sanction less effective bacteria that occupy a nodule. Sanctioning has been investigated in these studies by measuring a variety of factors, including rhizobial number, nodule size, nodule mass, bacteroid number, and internal nodule morphology. This has revealed that when the strain within a nodule fixes less or no N_2_ the plant sanctions this nodule by limiting its carbon supply, which results in reduced nodule size and bacterial abundance.

A previous study showed, by comparing the size of nodules and the carbon supply to these nodules, that peas apply sanctions to those less effective at fixing N_2_ by reducing their carbon supply [10]. These sanctions are not applied to nodules based on a set threshold value of fixation. Instead, peas sanction a nodule based on its N_2_-fixing ability relative to other nodules. Nodules containing mediocre fixing strains are sanctioned when other nodules containing highly effective fixing strains are present. These same nodules will be unsanctioned when the other nodules present contain poorly fixing strains. Sanctioning of nodules dependent on their bacterial strains’ relative fixation ability is called ‘conditional sanctioning’ [10]. It is likely that conditional sanctioning is dependent on the overall nitrogen and carbon status of the plant.

Conditional sanctioning raises important questions about the evolutionary stability of the symbiosis and the potential for exploitation by less cooperative strains. It has been shown that rhizobia of varying fixation effectiveness are present naturally within the rhizosphere [12, 13]; therefore, a strategy must exist through which less effective strains prosper. In this study, we investigated three avenues through which strains with reduced nitrogen fixation effectiveness might evade host sanctions and gain a competitive advantage. This involved testing the effects of three factors; the proportion of different strains, the distance between nodules containing different strains, and comparing strains with a smaller amplitude in variation of fixation effectiveness.

It has been proposed that mixed nodules, nodules containing multiple strains, may provide an avenue for mediocre strains to persist [5, 14–16]. However, other studies have proposed that mediocre strains are selectively sanctioned within mixed nodules [17, 18].

In natural settings, rhizobial populations are unlikely to infect hosts in a 1:1 ratio. As peas lack ‘partner choice’ (i.e. selecting more effective N_2_-fixing strains at the initial stage of infection and nodule formation) [11], they may be colonized by uneven ratios of effectively and ineffectively fixing strains. If most nodules are occupied by less effective strains, stringent sanctioning would threaten the plant’s nitrogen supply. However, if the plant relaxes sanctions to increase its nitrogen supply this would result in increased nodule size and greater abundance of the less effective strain. We tested this by inoculating peas with different ratios of a highly effective and a less effective strain and measured sanctioning.

Conditional sanctioning implies some form of comparison of the nitrogen output between nodules. Whether this occurs locally (between spatially proximate nodules) or globally (across the entire root system) remains unknown. If sanctions are applied based on local comparisons, a less effective strain might avoid sanctions by clustering with similar nodules. To assess this, we employed a split-root system to spatially separate strains and evaluate global versus local responses.

To date, studies have focussed on strains with relatively large differences in fixation capacity. For example, the Fix^+^ and Fix^int^ strains that were used in previous studies [10] vary by ~50% in fixation effectiveness. However, it remains unclear how finely tuned is the ability of plants to discriminate between strains. If the resolution is coarse, a strain with only slightly reduced fixation could avoid sanctions. To test this, we used a *fixL* double mutant [19], which fixes nitrogen at a level intermediate to the previously tested Fix^+^ and Fix^int^ strains [10]. This allowed us to assess the sensitivity of the sanctioning system of plants to subtle differences in symbiont performance.

Understanding how less effective strains might evade host control is crucial for explaining the evolutionary dynamics of the legume–rhizobia symbiosis and for maximizing the continued benefit of the symbiosis to modern agriculture. The scattered, paraphyletic distribution of nodulation within the nitrogen-fixing clade of rosids [20], including partial losses within Fabaceae, raises the possibility that uncontrolled exploitation of plant hosts by less effective rhizobia may contribute to the loss of nodulation in some lineages. It is important to consider that if the nitrogen-fixing symbiosis was engineered into cereal crops it would fail without some form of sanctioning.

In this study sanctioning was measured through the established method of comparing nodule size [8, 10, 11]. It has been shown that this method of measuring sanctioning leads to the same result as direct determination of carbon supply to nodules and is therefore a robust method for analysis [21]. Therefore, by comparing the size of nodules containing identical strains under different conditions (e.g. changing the coinoculant or changing the distance from nodules of different fixation capacities) we probed the effect of these factors on the carbon supply to nodules. This study will build on the work of previous studies, in particular previous work from this lab [10], by moving beyond the simple lab-specific 1:1 ratio of coinoculated strains with large variation in fixation ability. We instead test the spatial resolution of sanctioning as well as the ability of the host plant to discern between strains with small variations in fixation capacity. This study refines our understanding of host control mechanisms and their limitations, with implications for the stability and breakdown of mutualism in this important symbiosis.

## Materials and methods

### Rhizobial strains and culture conditions

The strains used in this work are all derived from an effective nitrogen-fixing bacteria *R. leguminosarum* bv. *viciae* Rlv3841, which is a root symbiont of *P. sativum* var. ‘Avola’ (pea) [22]. The strains used varied in their fixation ability or fluorescent tag but are otherwise isogenic (Table 1). The three near-isogenic strains used for this study are called Fix^+^, Fix^int^, and Fix^L^ [10, 19]. Fix^int^ contains a mutation within the promoter region of *nifA.* This insertion reduces the expression of *nifA*, which encodes the master transcription factor of nitrogen fixation*.* Fix^L^ is a double knock-out mutant for both copies of *fixL* in *R. leguminosarum*. FixL is essential for respiration in the microaerobic conditions within nodules. Some of the strains were tagged with a fluorescent marker [mCherry, green fluorescent protein (GFP)] to distinguish between nodules formed by each strain (Table 1). Strains were maintained on tryptone–yeast (TY) agar with the required concentrations of antibiotics (Table 1) [23]. For longer-term storage a solution of TY with 15%–20% glycerol was inoculated and then stored at −80°C. For inoculation of peas rhizobia were grown on a TY agar slope. The slopes were then washed using universal minimal salts medium (UMS) [24]. The number of bacteria present in the medium was determined by measuring the OD_600_ using a Genesys 250 UV-Visible Spectrophotometer and cells diluted to ~1 × 10^7^ CFU ml^−1^.

**Table 1:** Rhizobial strains; all strains derived from Rlv3841 and provided with a strain code, resistance markers, short description, and reference.

Name	Strain	Antibiotic resistance	Description	Reference
Fix^+^	Rlv3841	Streptomycin	Rlv3841	[22]
Fix^+^ (GFP)	OPS1339	Streptomycin, gentamicin	Rlv3841 Tn7-Gm- GFP	[10]
Fix^int^ (mCherry)	OPS2269	Streptomycin, spectinomycin, gentamicin	Fixint Tn*7*-Gm- mCherry Rlv3841 mutant, Ω Spc cassette in promoter region of *nifA*	[10]
Fix^L^	LMB496	Streptomycin, spectinomycin	hfixL_9_:: omega spec, hfixL_c_:Pk19	[19]

### Plant growth

Pea seeds were surface-sterilized (1 minute in 95% ethanol followed by 5 minutes in 2% NaClO), rinsed with sterile water, and left to germinate for 5 days on 1% w/v agar plates at room temperature in the dark. Plants were grown in a growth chamber (21°C, 16 h photoperiod). The growth chamber lights provided a 180 μmol m^−2^ s^−1^ photosynthetic photon flux density.

#### Single-pot experiments

After 5 days seedlings were transplanted to a sterilized 500 ml Azlon beaker for competition assays and a 1 l Azlon beaker for acetylene reduction assays. The beakers contained a 1:1 mixture of silver sand and fine vermiculite, and a sterilized nitrogen-free nutrient solution (75 ml for 500 ml beakers and 150 ml for 1 l beakers) [11]. For single-strain experiments 1 × 10^7^ CFU of the desired rhizobial strain was added, mixed with the 75 ml or 150 ml of the nutrient solution. For coinoculated experiments the desired ratio (1:9, 1:2, 1:1, 2:1, or 9:1) of the two rhizobial strains was added, with the combined number kept at 1 × 10^7^ CFU (see Westhoek *et al.* [11]). The beakers were covered with cling film to prevent aerial contamination. This was slit after a few days to allow seedlings to grow through. Plants were grown for 28 days and watered as necessary from 7 days onwards.

#### Split -root experiments

After 5 days the primary root of the seedling was cut laterally using a sterile scalpel, removing the tip of the root below where lateral root hairs had begun to form. These cut seedlings were then moved onto fresh 1% w/v agar plates; the edges of the plates were sealed with Micropore tape and then placed in a growth chamber (21°C, 16 h photoperiod) for a further five days. Seedlings were then placed across two 500 ml Azlon beakers with approximately half of the newly formed lateral roots placed in each beaker. The beakers contained a 1:1 mixture of silver sand and fine vermiculite and 75 ml of sterilized nitrogen-free nutrient solution. To each pot a total of 0.5 × 10^7^ CFU of the desired strain/strains was added in the 75 ml of nutrient solution. The exposed root split across the pots was lightly watered every day for the first 7 days and then watered as necessary. In split-root experiments a 1:1 ratio of each strain was always applied to the plant either evenly mixed across both roots or with one strain on one side and the second strain on the other.

### Harvesting

Plants were harvested 28 days postinoculation (dpi). Plants were removed from the sand vermiculite mix and washed carefully. Nodules were then imaged using a LEICA M165 FC fluorescent stereo microscope and an iBright FL1500 imaging system. Nodule occupants were identified based on their fluorescence (Fix^+^ tagged with GFP and Fix^int^ tagged with mCherry) or the lack of fluorescence (Fix^L^ untagged). The nodules of each strain were counted either per plant for single-beaker experiments or per beaker for split-root experiments.

### Nodule measurement

Measurements were taken of the five largest nodules of each strain, on each plant, or the side of the split root. The five largest nodules were selected so as to eliminate variation due to the developmental stage of the nodules as nodules of all the strains would be much smaller at early stages of development. The nodules were selected by eye and then picked and photographed. These nodules were then measured using the Fiji analysis software (v2.14.0/1.54f). A ruler was included in each photograph of the nodules so that the scale for each image could be set by drawing a line of 10 mm over the markings of the ruler using a straight-line selection. Each nodule was then drawn around using the polygon selection tool and the area of the region of interest was measured. The mean of the five nodule sizes was calculated and taken as the size of the developed nodules containing a specific strain.

### Acetylene reduction assays

For acetylene reduction assays whole plants were placed directly into 250 ml Schott bottles; the total volume of these bottles, including the headspace, was 320 ml, with airtight neoprene seals. Acetylene gas was fed through a rubber tube and taken up via a syringe to be added to the Schott bottle to make up 2% of the bottle’s volume (6.4 ml). This low concentration was used to minimize the effect of the acetylene on the nitrogenase [25]. The acetylene reduction assay was then carried out using the method described in previously published literature [10]. The fixation rate was calculated through gas chromatography based on the fraction of the acetylene gas, 2% of the total volume in the Schott bottle, converted to ethylene in the space of 1 h divided by the total number of nodules. The samples were measured immediately after 1 h and the fixation effectiveness was calculated as a percentage of the average fixation per hour per nodule of Fix^+^.

### Statistical analysis

#### Single-pot experiment, peas coinoculated with Fix ^+^ and Fix^int^

To test the correlation between the proportion of a strain within the inoculum and the proportion of nodules containing the strain linear regression analysis was employed. Linear regression was also used to assess the correlation between the number of nodules a strain occupied and the size of the nodules containing that strain.

#### Split-root experiment, peas coinoculated with Fix ^+^ and Fix^int^

When testing for the difference in size between Fix^+^ and Fix^int^ nodules in a split-root system, a nested linear mixed-effects model was used in which the experiment number was a random factor and the plant number was used as a grouping factor allowing a pairwise comparison of nodules on the same plant across our biological replicates. As in the single-pot experiments, when assessing the correlation between the number of nodules a strain occupied and the size of those nodules, linear regression was used.

To test for significant differences in the size of nodules on a split-root plant when inoculated into separate pots or mixed into both pots, a nested linear mixed-effects model was used with the experiment number as a random factor and the plant as a grouping factor for pairwise comparison. This model was then tested using a one-way ANOVA; if the ANOVA showed a significant difference between groups, this was then followed up with Tukey’s *post hoc* test.

#### Single-pot experiment, peas coinoculated with Fix ^+^, Fix ^int^, Fix ^L^ in pairwise combinations

To test the effect of the coinoculum on the three strains of varying fixation effectiveness, a linear model was produced for each strain comprising the size of the nodules containing the strain and the coinoculum (either one of the other two strains or the same strain). A one-way ANOVA was then used to test whether the coinoculum led to a significant variation in nodule size. If this test returned a significant difference, then Tukey’s *post hoc* test was used to compare each of the coinocula to the others.

All the statistical analyses were carried out using R version 4.4.1 (2024-06-14) and RStudio (2022.02.3 + 492 ‘Prairie Trillium’). Graphs were produced using GraphPad version 9. An R markdown document is provided (Fig. S1). The model assumptions of equal variance and normality were assessed by visual inspection of residual plots (see R markdown). All the statistical test outputs are provided in the R markdown (Fig. S1). Data for experiments are provided (Table S1).

## Results

### Sanctioning severity is dependent on the proportion of less effective strains

**Figure 1:**
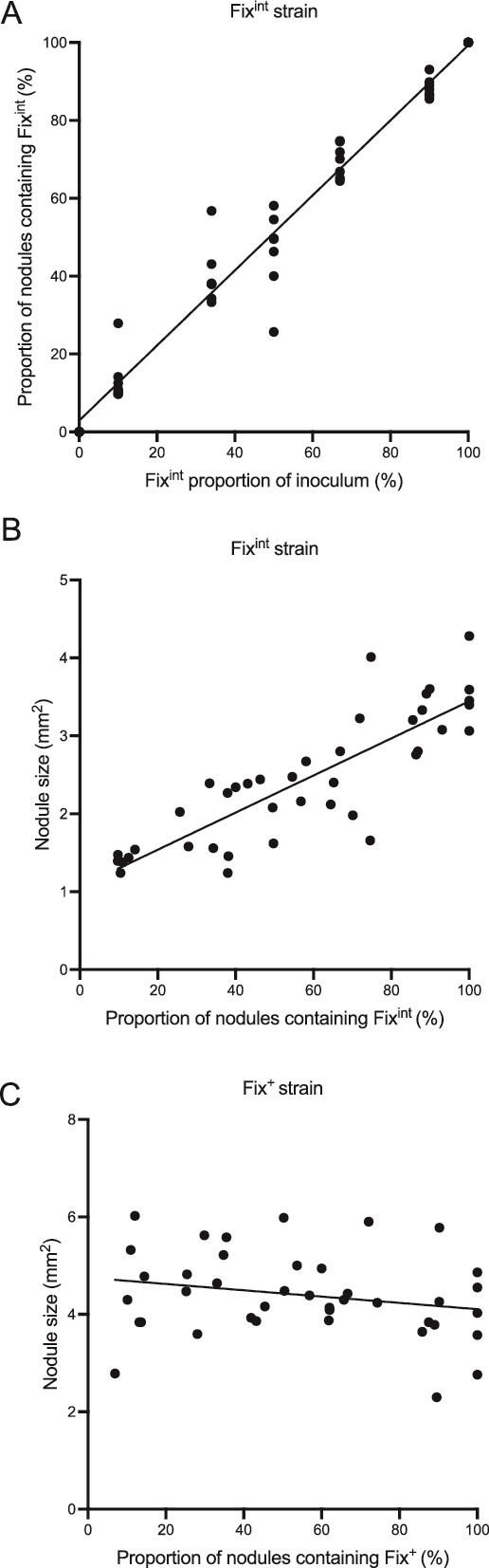
The size of less effective nodules is dependent on their proportion of total nodules. Peas were inoculated with fluorescently tagged Fix^+^ (GFP) and Fix^int^ (mCherry) strains at varying ratios. The percentage of nodules containing a Fix^int^ strain and the percentage of nodules containing a Fix^int^ strain within the inoculum applied was measured and compared (A). The size of the nodules containing Fix^int^ (B) and of those containing Fix^+^ (C) on these plants was plotted against the percentage of nodules containing the relevant strain. Each point represents the percentage (A) or the size (B and C) for one plant. The trend line is a linear regression.

To test the effect of varying the frequency of the less effective strain, peas were inoculated with Fix^+^ (GFP) and Fix^int^ (mCherry) at varying ratios. There was a significant linear correlation between the proportion of the inoculum and the proportion of nodules occupied by the strain (*y* = 0.99*X* − 1, *R*^2^ = 0.96, *P* < .0001) (Fig. 1A). The size of the nodules containing the less effective Fix^int^ was significantly positively correlated with the frequency of nodules containing Fix^int^ (*y* = 30*X* − 16, *R*^2^ = 0.73, *P* < .0001) (Fig. 1B) and approached, but did not meet, the size of Fix^+^ nodules as they dominated nodulation. In contrast, the size of the nodules containing the more effective Fix^+^ strain did not show a significant correlation to the number of nodules the strain occupied (*y* = −8.02*X* − 90, *R*^2^ = 0.03, *P* = .16) (Fig. 1C).

### Sanctioning is based on a global comparison between nodules

To test to what extent sanctioning is controlled through a global or local signalling mechanism, sanctioning was tested in a split-root system with different strains in physically separate vessels, thereby eliminating any potential effect of a local signalling mechanism between nodules that were proximal to each other.

**Figure 2:**
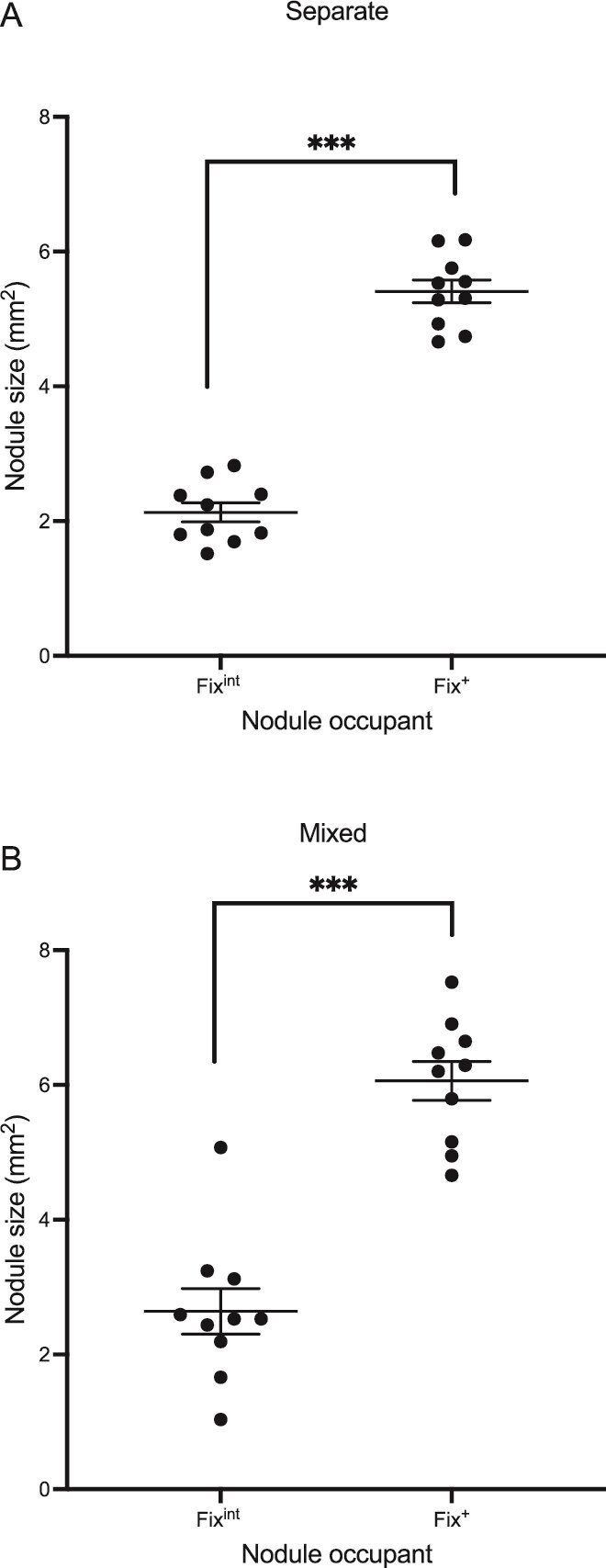
In split-root peas, with separated inocula, less effective nodules were smaller than more effective nodules. Peas with split roots placed across two pots were inoculated with a Fix^+^ (GFP) strain and a Fix^int^ (mCherry) strain, with each strain either added to a separate pot (A) or added to both pots in a 1:1 ratio. The size per plant of the nodules for each strain was measured. Each point indicates the average value for a single plant. Statistical comparison carried out through paired *t*-test. ^***^P < .001.

When spatially separated on different roots, Fix^+^ nodules were significantly larger than Fix^int^ nodules (est. = 3.42, SE = 0.17, df = 9, *t* = 19.91, *P* < .0001) (Fig. 2A). This result demonstrates that the effect of sanctions is not altered by the proximity of the nodules across a split-root system. Therefore, legumes carry out a global comparison between the nodules present and apply sanctions accordingly.

### Proximity does not alter sanctioning

To explore whether there is a local as well as a global comparison of nodule outputs, a series of split-root plants were inoculated with both strains in both vessels.

The Fix^int^ nodules on split roots with mixed inoculum were significantly smaller than the Fix^+^ nodules on the same plants (estimate = 3.28, SE = 0.16, df = 8.99, *t* = 19.98, *P* < .0001) (Fig. 2B). When comparing the nodules on the separately inoculated split roots to those on the mixed inoculated plants, there was no significant difference between the two sets of Fix^+^ nodules (estimate = 0.51, 95% CI = −0.43 to 1.46, *P* = .47) or the two sets of Fix^int^ nodules (estimate = 0.65, 95% CI = −0.30 to 1.60, *P* = .27). In short, the results of the mixed inoculated split-root plants were identical to those of the separately inoculated split roots.

### Peas can detect small changes in fixation effectiveness

**Figure 3:**
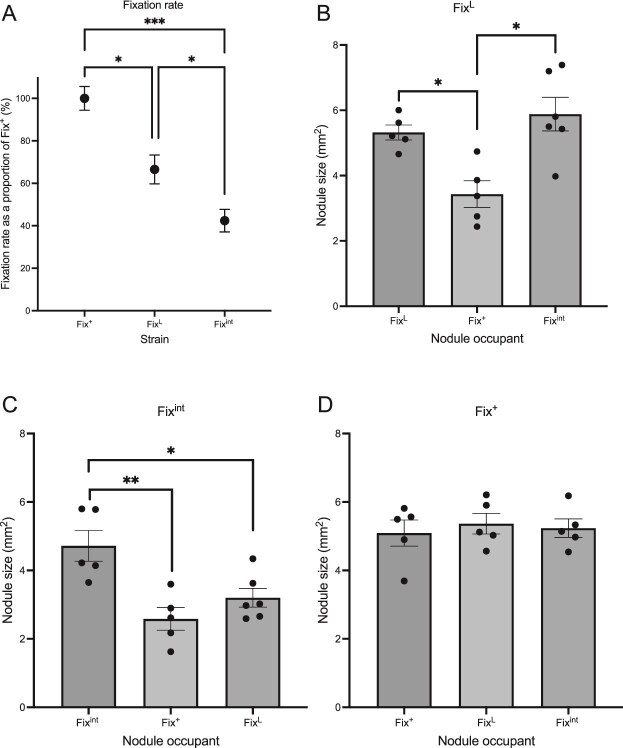
In pairwise comparisons Fix^L^ and Fix^int^ nodules were smaller when coinoculated with a more effective strain. The rate of fixation was measured for three strains on solo-inoculated pea plants. The values were converted to a percentage of the average fixation rate of Fix^+^ (wild-type Rlv3841) (A). Black dots denote average fixation effectiveness. Error bars are one SE of the mean. Peas were inoculated with a combination of a Fix^L^ (untagged) (B), Fix^int^ (mCherry) (C), and a Fix^+^ (GFP) (D) strain. These strains were coinoculated either with themselves or with one of the other two strains. Nodules were harvested after 28 days and the size of fully developed nodules was measured. Each black point indicates the nodule size for one plant. Bars indicate the mean of the nodule sizes. Error bars indicate the standard error of the mean. Statistical comparison carried out through one-way ANOVA and Tukey’s *post hoc* test. ^*^P < .05, ^**^P < .01, ^***^P < .001.

To test the ability of peas to detect small differences in fixation effectiveness, plants were inoculated with one of three combinations of three isogenic strains, the Fix^+^ and Fix^int^ strains as well as the Fix^L^ strain. Within pea nodules the Fix^L^ strain fixes at ~67% of Fix^+^, the Fix^int^ strain fixes at ~43% of the Fix^+^ strain (Fig. 3A), and therefore the difference in fixation between the Fix^L^ strain and the Fix^+^ strain is 33% and between the Fix^L^ strain and the Fix^int^ strain it is 24%. Therefore, the difference in fixation rate between Fix^L^ and either of these strains is smaller than the difference in fixation rate of the Fix^+^ and Fix^int^ (57%) strains used in previous sanctioning studies.

When Fix^L^ was coinoculated with Fix^+^, the nodules containing Fix^L^ were significantly smaller than when Fix^L^ was the sole inoculant (estimate = −1.90, 95% CI = −3.52 to −0.26, *P* = .02). There was no significant difference in the size of nodules containing Fix^L^ when coinoculated with Fix^int^ compared to when Fix^L^ was the sole inoculant (estimate = −0.56, 95% CI = −2.12 to −1.00, *P* = .62) (Fig. 3B).

Compared to when Fix^int^ was the sole inoculant, nodules containing the Fix^int^ strain were significantly smaller when coinoculated with either strain: Fix^+^ (estimate= −2.14, 95% CI = −4.01 to −0.89, *P* = .003) or Fix^L^ (estimate= −1.52, 95% CI = −2.81 to −0.23, *P* = .02) (Fig. 3C).

Fix^+^ nodules did not significantly vary in size regardless of the coinoculant or when inoculated solo (df = 2, *F* = 0.18, *P* = .84) (Fig. 3D).

## Discussion

The existence of host sanctions is now a well-established concept within the field [7, 10, 11, 26]. However, this study sought to address a crucial unresolved question. How does a host plant manage sanctions when factors such as the ratio of strains, their proximity to one another, and their relative fixation ability vary? In this study we have tested several potential mechanisms by which a rhizobial strain of lower fixation ability could evade host sanctions.

**Figure 4:**
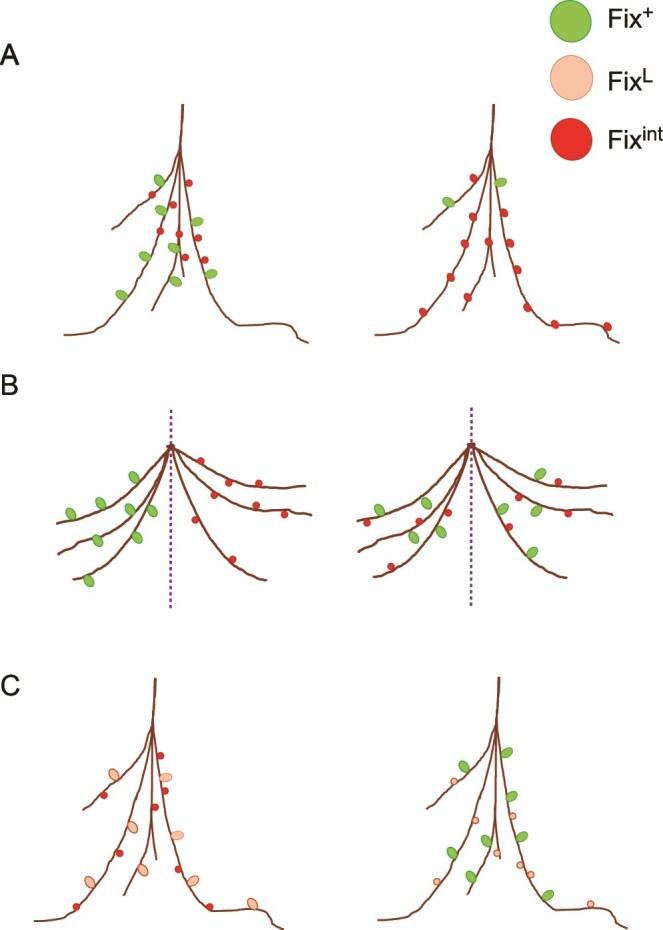
Conclusions of study on proportion, proximity, and sensitivity in sanctioning. Plants were inoculated with pairwise combinations of strains: Fix^+^ (GFP), Fix^L^ (untagged), and Fix^int^ (mCherry). When the proportion of the less effective strain was increased, the less effective nodules were larger (A). Split-root peas (split indicated by dashed line) spread across two separate vessels were inoculated with a more and a less effective strain. Regardless of the distance between nodules of differing fixation ability, the less effective nodules were significantly smaller (B). When Fix^L^ was introduced as a strain fixing at a level between Fix^+^ and Fix^int^, the pea was still able to discriminate between the strains and Fix^L^ nodules were smaller when coinoculated with Fix^+^ (C).

We have shown that peas moderate the severity of the sanctions imposed on a nodule containing a less effective bacterial strain depending on the proportion of nodules occupied by this strain (Fig. 1). Therefore, when occupying a majority of nodules a less effective strain may evade sanctioning. We also conclude that peas compare nodules across their root system (Fig. 2A) and this does not include a local comparison between nodules that are proximal to each other (Fig. 2B). This could be via a direct comparison of nodule ammonia output. It could instead be that when the ammonia output for a nodule exceeds a threshold value based on the overall carbon and nitrogen status of the plant (including the output of all the other nodules) sanctions are not applied. We have tested the ability of peas to differentiate between smaller variations in fixation effectiveness than variations that have been previously tested for and found that they were still able to sanction the less effective strain (Fig. 3). However, this result does not allow us to make a broader conclusion on the ability of a less effective strain with a smaller variation in fixation effectiveness to evade sanctions. Therefore, we conclude that conditional sanctioning in peas is dependent on the proportion of different strains, is not affected by proximity between nodules, and can differentiate between small variations in fixation effectiveness of bacterial strains (Fig. 4).

This study has tested various avenues by which a less effectively fixing strain could prosper within a population of nitrogen-fixing bacteria. We found that if a less effective strain dominates nodulation, then this strain will not be sanctioned. This is likely because when such a large number of nodules are occupied by a less effective strain the plant is unable to meet its nitrogen requirements by only rewarding the more effective strain [26]. As a result, the host plant must reduce or entirely suspend sanctioning of nodules containing the less effectively fixing strain.

The lack of sanctions when less effective strains occupy most nodules may explain why sanctions have failed to eradicate less effective strains in wild populations [12, 13]. Therefore, when considering the use of an effectively fixing inoculum to boost yields it is crucial to consider the relative population size and competitiveness of a strain compared to the pre-existing strains in the soil. If too few nodules are inhabited by the introduced strain, then it is likely be outcompeted by strains already present even if they are less effective at fixing N_2_.

The possibility of a less effective strain overcoming sanctioning to outcompete a more effective strain may provide evidence for the multiple-loss theory for the paraphyletic distribution of the symbiosis [27]. Therefore, when a less effective strain successfully evades sanctions, it results in the collapse of the symbiosis as the interaction is no longer beneficial for the host plant. In this way individual symbioses may be lost in a manner analogous to the ‘Red Queen’ hypothesis [28], in which both organisms must continually adapt to maintain the stability of the interaction.

From our results, we hypothesize that a first step in sanctioning is the plant determining its global nitrogen status. This would then be compared to the nitrogen output of each individual nodule. Our results support this as we have shown that plants carry out sanctioning of individual nodules based on comparison to all the nodules across the root. Given the variation in sanction severity based on the proportion of nodules occupied by a strain, it seems the plant mediates sanctions based on the number of nodules present, their fixation rates, and the plant’s nitrogen requirements. In short, the plant assesses how many nodules it needs to invest in to meet its nitrogen requirements. Our results show not only this but also that the comparison between nodule fixation rates occurs at a finer resolution than previously shown. This detection and integration of information then results in the sanctioning of individual nodules.

A potential mechanism may require a signalling system that can respond to nodule outputs and then trigger developmental changes in the root architecture based on comparative nodule ability. Candidate signalling molecules therefore include plant hormones, e.g. abscisic acid (ABA). ABA could, theoretically, act as a negative regulator of nodule sanctions as its presence inhibits ABI1, which is a negative regulator of SnRK1, which is required for malate production in nodules [29, 30]. The role of candidate hormones may be tested through hormone biosynthesis mutants such as the ABA biosynthesis mutant *wilty* [31]. The loss of effective sanctioning in such a mutant would provide clear evidence for a regulatory role in sanctioning.

Future studies should therefore attempt to identify such a signalling system, and any signalling molecules involved. It might be hypothesized that the sanctioning signalling pathway will be underpinned by a genetic regulatory one. Therefore, future studies may focus on identifying such a pathway. This may be achieved through RNA sequencing of sanctioned and unsanctioned nodules and then using differential expression analysis to identify genes that are significantly altered in their expression due to sanctioning. Alternatively, the screening of a *P. sativum* TILLING population for mutants defective in sanctioning would also be a viable route to identify essential genes for sanctioning [32].

It could be hypothesized that the phenomenon observed is not the result of active sanctioning and instead results from a simple source–sink system. The energetically expensive process of nitrogen fixation would create a sink for carbon within the nodule relative to the amount of N_2_ fixation in a manner analogous to the previously described feedback system between plant nitrogen demand and carbon allocation to nodules [33]. A nodule with higher fixation will be a bigger sink and will receive more carbon. Such a system would be entirely centred around the consumption of energy rather than the production of ammonia. However, while theoretically this might enable an inefficient strain to evade sanctions it is inconsistent with previous work [7, 8, 11]. This hypothesis would also, potentially, select for cheating strains. This is because a strain that expends large quantities of energy on replication while fixing little or no nitrogen would be rewarded in such a source–sink system.

This study demonstrates the complexity of the regulatory mechanism behind the application of host sanctions in the pea–*Rhizobium* symbiosis. It is clear that the host plant in this symbiosis can integrate large quantities of information regarding the nitrogen output of the individual nodule and the global nitrogen status (including the output of the other nodules within the root system) before making individualized decisions on the sanctioning fate of a nodule. This system appears to be critical to the stability of this symbiosis and therefore, a fundamental understanding of sanctioning is critical to the successful utilization of biological nitrogen fixation for sustainable agriculture.

## Supplementary Material

Supplementary_Material_wrag097

## Data Availability

All the data generated or analysed during this study are included in this published article (and its online supplementary material).
